# Combining Bioinformatics Techniques to Study Diabetes Biomarkers and Related Molecular Mechanisms

**DOI:** 10.3389/fgene.2020.00367

**Published:** 2020-04-30

**Authors:** Han Nie, Kaihua Zhang, Jiasheng Xu, Kaili Liao, Weimin Zhou, Zhonghua Fu

**Affiliations:** ^1^Department of Vascular Surgery, The Second Affiliated Hospital of Nanchang University, Nanchang, China; ^2^Department of General Surgery, Jiujiang Hospital Affiliated to Nanchang University, Nanchang, China; ^3^Department of Clinical Laboratory, The Second Affiliated Hospital of Nanchang University, Nanchang, China; ^4^Department of Burns, The First Affiliated Hospital of Nanchang University, Nanchang, China

**Keywords:** miRNA-126, miRNA-28-3p, diabetes mellitus, biomarkers, bioinformatics analysis, circulating miRNA

## Abstract

**Objective:**

To explore the mechanism of plasma circulating miRNA-126 and miRNA-28-3p in diabetes mellitus (DM) patients, and to identify the related bioinformatics analysis.

**Methods:**

Randomly selected 120 DM patients as the observation group and 120 non- DM patients as the control group. The plasma circulating miRNA-126 and miRNA-28-3p were analyzed by qRT-PCR, and their target genes, biological information, related lncRNA and circRNA were predicted.

**Results:**

The circulating miRNA-126 (0.1162 ± 0.0236 vs. 0.0018 ± 0.0862) and miRNA-28-3p (0.1378 ± 0.0268 vs. 0.0006 ± 0.0167) levels in the observation group were significantly higher than those in the control group, and differences were statistically significant (*P* < 0.01). The Pearson correlation coefficient of miRNA-126 and miRNA- 28-3p was 0.4337 (*P* < 0.01). ROC curve analysis of miRNA-126 and miRNA-28-3p showed that the differences of the area under curve were statistically significant between the two groups (*P* < 0.01). Bioinformatics prediction showed that miRNA-126 and miRNA-28-3p may be involved in regulation of the insulin signaling pathway, insulin receptor signaling pathway, insulin/insulin growth factor signaling pathway, mitogen-activated protein kinase (MAPK) signaling pathway and angiogenesis. Moreover, it may be associated with a variety of lncRNA and cir-cRNA.

**Conclusion:**

Circulating miRNA-126 and miRNA-28-3p can be a potential biomarker of DM and it may play an important role in the DM by regulating insulin or insulin growth factor related signaling pathways.

## Introduction

Diabetes mellitus (DM) is one of the common clinical diseases in the world. It is a group of metabolic diseases characterized by chronic hyperglycemia and one of the most important chronic non-communicable diseases. The prevalence of DM in adults aged 18 years and older is 11.6%, and the majority of them are type 2 diabetes mellitus (T2DM) (Nichols et al., 2014; Mozaffarian et al., 2016; Zhou et al., 2016). Due to the acceleration of urbanization, an aging population, the change in lifestyle (significant reduction in physical activity, the marked increasing in the proportion of fat intake, and the increasingly fast the pace of life), the increase in the proportion of obesity, overweight, and other factors, the prevalence of DM may be higher and higher, and there are more patients with DM; DM has a higher morbidity and mortality, and is an independent risk of atherosclerotic vascular disease. It is one of the factors that have brought a serious burden on the health of individuals and society (Eckel et al., 2011; Zeitler et al., 2014; Garber et al., 2015). Prevention and treatment of DM is the main solution to reduce mortality, disability and improve quality of life. With the application of hypoglycemic drugs, the popularization of blood glucose monitoring methods, the control of complications of DM, and lifestyle interventions, DM has achieved certain prevention and treatment. As a result, its direct mortality rate has declined; however, due to the limited epidemiological survey status, the high proportion of undiagnosed population, and a large number of DM high-risk groups (Zeitler et al., 2014; Harper et al., 2015; Garber et al., 2015), the prevention and treatment of DM has yet to be further studied. Recent studies have shown that RNA (microRNA, miRNA) is closely related to the pathogenesis of DM, and miRNA-126, miRNA-28-3p, and miRNA-423-5p may be involved in the pathogenesis of DM (Mediavilla Bravo, 2014; Feng et al., 2016). MicroRNAs may be involved in the development of a variety of cardiovascular diseases, such as atherosclerosis, myocardial infarction, heart failure, hypertension, pulmonary hypertension, arrhythmia and cardiomyopathy, etc. (Chen and Can, 2015; Cheng et al., 2015; Beermann et al., 2016); Some studies have also found some serum or plasma. The circulating miRNA can be used as a potential disease diagnostic biomarker for DM (Busch et al., 2016; Navickas et al., 2016), providing new ideas and new methods for the prevention and treatment of DM. However, there are few clinical samples of plasma circulating miRNA-126, miRNA-28-3p and DM. Therefore, this paper intends to investigate the relationship between plasma circulatory miRNA-126, miRNA-28-3p and DM, and conduct related bioinformatics analysis in order to explore new ways for DM prevention and provide theoretical support.

## Materials and Methods

### Clinical Data

Patients were selected from September 2016 to May 2018 in the Department of Endocrinology, Internal Medicine Division, The Second Affiliated Hospital of Nanchang University.

Eighty patients with T2DM were included in the observation group (DM group), and 120 patients without DM who were hospitalized in the endocrinology department and the cardiology department were used as the control group. The diagnostic criteria of T2DM are diagnosed and classified using the criteria for DM diagnosis, glucose metabolism status classification, and DM classification system recommended in the “CONSENSUS STATEMENT BY THE AMERICAN ASSOCIATION OF CLINICAL ENDOCRINOLOGISTS AND AMERICAN COLLEGE OF ENDOCRINOLOGY ON THE COMPREHENSIVE TYPE 2 DIABETES MANAGEMENT ALGORITHM–2016 EXECUTIVE SUMMARY.” Patients with malignant tumors, acute infections and systemic immune diseases, thyroid diseases, various organ transplants, and severe heart, lung, liver, and renal insufficiency were excluded. Patients in the two groups were equal in age, sex, and body mass index. There was no statistically significant difference in the basic medical history of dex, BMI, smoking history, coronary heart disease, hypertension, and stroke (*P* > 0.05); in conventional cardiovascular medications such as beta-blockers, calcium channel blocker (CCB).

There was no statistically significant difference between CCB, angiotensin converting enzyme inhibitor (ACEI), and angiotensin receptor blocker (ARB) (*P* > 0.05); There was no significant difference in biochemical examination between TC and other patients (*P* > 0.05), such as serum creatinine (Scr), blood urea nitrogen (BUN), glucose (Glu), total cholesterol (TC), and total cholesterol (total cholesterol). The average age of DM patients in the DM group was 5.246 years. The average fasting blood glucose was 12.02 mmol/L, and the postprandial blood glucose was 15.66 mmol/L in average; the difference between serum uric acid (SUA), lipoprotein (a), lipoprotein (a), Lp (a), high density lipoprotein, and HDL was not statistically significant (*P* > 0.05). The low-density lipoprotein (LDL) in the control group was slightly higher than that in the DM group (*P* < 0.05), as shown in Table 1.

**TABLE 1:** Basic medical history and biochemical examination of two groups of patients (*n* = 120).

Item	DM group	control group	*P*
Age (years)	67.81 ± 1.11	64.95 ± 1.19	0.0797
Sex (male/female, case)	43/37	45/35	0.7506
BMI (kg/m^2^)	23.00 ± 0.20	25.35 ± 2.20	0.2978
History of smoking (case)	35	37	0.7506
Coronary heart disease (case)	25	28	0.6143
Hypertension (case)	34	37	0.6473
Atrial fibrillation (case)	17	15	0.6926
Stroke (case)	16	13	0.5381
β-receptor blockers (case)	35	36	0.8736
CCB (case)	30	22	0.4933
ACEI (case)	25	27	0.8661
ARB (case)	28	22	0.3939
SUA (μmol/L)	328.22 ± 10.92	314.12 ± 13.47	0.4126
SCR (μmol/L)	89.00 ± 6.42	96.40 ± 12.64	0.6022
Duration of DM in patients (years)	5.25 ± 1.91	–	–
Maximum fasting glucose (mmol/L)	12.02 ± 0.60	–	–
Postprandial blood glucose (mmol/L)	15.66 ± 0.71	–	–
Glu (mmol/L)	5.56 ± 0.21	5.99 ± 0.16	0.1029
Lp(a) (mmol/L)	204.82 ± 17.89	183.43 ± 17.67	0.3993
BUN (mmol/L)	4.90 ± 0.23	8.88 ± 2.92	0.1754
TC (mmol/L)	4.90 ± 0.12	5.14 ± 0.17	0.2400
TG (mmol/L)	1.55 ± 0.08	1.43 ± 0.11	0.3630
HDL (mmol/L)	1.43 ± 0.04	1.35 ± 0.04	0.1443
LDL (mmol/L)	2.75 ± 0.11	3.09 ± 0.14	0.0485

### Sample Collection

All selected patients provided 2 mL of venous blood with EDTA anticoagulant tubes within 4 h of admission, and then centrifuged (2500 r/min, 4°C, 10 min) in 2 h to separate plasma and dispensed in 1.5 mL EP tubes. Stored in a refrigerator at 120°C for follow-up experiments; collected basic medical history, medications for cardiovascular disease, and basic biochemical tests.

### Plasma miRNA-126 and miRNA-28-3p Real-Time Fluorescent Quantitative Detection

The miRNA was extracted and purified by column isolation using miRNAcute miRNA Extraction and Separation Kit (Shanghai MiTuo Biotechnology Co., Ltd.), and then miRNA was reverse-transcribed to cDNA according to its companion miRNAcute miRNA cDNA first-strand synthesis kit (Shanghai MiTuo Biotechnology Co., Ltd.). Finally, fluorescence quantitative detection was performed according to the miRNAcute miRNA SYBR Green Fluorescent Quantitative Detection Kit (Guangzhou Zhenzhi Biotech Co., Ltd.) and downstream primers in the kit. hsa-miR-126-3p (hsa-miR-126, MIMAT0000445 UCGUACCG UGAGUAAUAAUGCG), hsa-miR-28-3p (MIMAT0004502 CACUAGAUUGUGAGCUC-CUGGA).

The upstream primers and internal reference U6 primers of hsa-miR-126-3p and hsa-miR-28-3p were provided by Shanghai MiTuo Biotechnology Co., Ltd. (shanghai, China). The downstream primers were universal primers for the kit. All procedures were performed according to the kit instructions. The fluorescence quantification procedure was performed at 94°C for 2 min; the template was denatured at 94°C for 20 s in the PCR cycle; annealing was performed at 60°C for 34 s; a total of 30 cycles. In this study, 2 – ΔCt relative quantification method was used for data analysis: ΔCt (experimental group) = Ct (experimental group target gene) − Ct (experimental group reference gene); ΔCt (control group) = Ct (control group target gene) - Ct (control group internal reference).

### miRNA-126 and miRNA-28-3p Bioinformatics Prediction

Target bioinformatics software such as targetScan, GCBI online website, microRNA.org, and miRanda was used to predict the target genes of miRNA-126 and miRNA-28-3p; KEGG pathway and starBase were used to predict the basic bioinformatics function, the signaling pathways that may be involved in regulation, the related circular RNAs (circular RNA, circRNA) and long non-coding (lncRNA), etc. (Li et al., 2014), and analyzed and mapped the results using CircNet software (Liu et al., 2016); Using miRNAMap2 to predict miRNA-126 and miRNA-28-3p expression levels of various organs (Hsu et al., 2007); UCSC Genome Browser was used to predict introns, exons and polymorphic sites of miRNA-126 and miRNA-28-3p (Mulligan et al., 2017; Tyner et al., 2017).

### RT-qPCR Detection of the Target Genes in Plasma

RT-qPCR was used to verify the expression level of the most relevant five target genes regulated respectively by miRNA-126 and miRNA-28-3p in serum. The primers for the top five most relevant target genes regulated by miRNA-126 and miRNA-28-3p, respectively, are shown in Table 2 (Primers were synthesized according to the PCR primer information provided by the Primer Bank database). The real-time PCR kit was used to detect the expression of these genes and to draw statistical charts. The reaction procedure was: Hol d (pre-denaturation): 95°C, 30 s, 1 cycle; Two-step PCR: 95°C, 5 s, 60°C, 30 s, 40 cycles; Dissociation: 95°C, 15 s, 60°C, 30 s, 95°C, 15 s, 1 cycle. GAPDH was used as an internal reference and a two-step method was used. The expression of GAPDH was detected by qPCR. Using the expression level of GAPDH as the standard value “1,” the relative expression levels of each target gene in the plasma of DM and control group were calculated.

**TABLE 2:** The primers for the top five most relevant target genes regulated by miRNA-126 and miRNA-28-3p, respectively.

	Sequence (5′–>3′)		Amplicon Size	Corresponding microRNA
VEGFA	Forward	AGGGCAGAATCATCACGAAGT	75	miRNA-126
	Reverse	AGGGTCTCGATTGGATGGCA		
SPRED1	Forward	GAGGGAGTGGACTAAGCAGC	241	miRNA-126
	Reverse	CCTCTATCAAAAGCCCTAGCATC		
PIK3R2	Forward	AAAGGCGGGAACAATAAGCTG	85	miRNA-126
	Reverse	CAACGGAGCAGAAGGTGAGTG		
PARP16	Forward	CCTCAAAGGTCCTGACAATCC	198	miRNA-126
	Reverse	CTAGGCGGCTACCATGAAATG		
SLC7A5	Forward	GGAAGGGTGATGTGTCCAATC	83	miRNA-126
	Reverse	TAATGCCAGCACAATGTTCCC		
IGF1	Forward	GCTCTTCAGTTCGTGTGTGGA	133	miRNA-28-3p
	Reverse	GCCTCCTTAGATCACAGCTCC		
IGF2R	Forward	GTGACCAGCAAGGCACAAATC	245	miRNA-28-3p
	Reverse	CACCAAGTAGGCACCACTAAG		
MAP2K3	Forward	GAGGGAGACGTGTGGATCTG	129	miRNA-28-3p
	Reverse	CCGCACGATAGACACAGCAAT		
MAPK1	Forward	TCTGGAGCAGTATTACGACCC	134	miRNA-28-3p
	Reverse	CTGGCTGGAATCTAGCAGTCT		
RAF1	Forward	GGGAGCTTGGAAGACGATCAG	165	miRNA-28-3p
	Reverse	ACACGGATAGTGTTGCTTGTC		

### Statistical Analysis

Data in GraphPad Prism 7 (GraphPad Software Inc., CA, United States) and IBM SPSS 17.00 (IBM Analytics, United States) were used for analysis. Qualitative data were analyzed using χ^2^-test or Fisher test. Measured data were expressed as mean ± standard deviation (x ± s) and data were compared using t test. The receiver operating characteristic curve (ROC curve) and ROC analysis were completed by GraphPad Prism 5. *P* < 0.05 was defined as statistically significant, *P* < 0.01 was defined as very significant statistical significance.

## Results

### Expression of Plasma miRNA-126 and miRNA-28-3p

**FIGURE 1:**
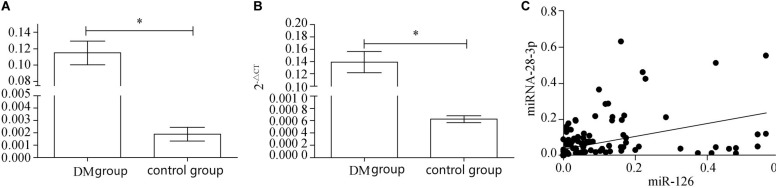
**(A)** Real-time fluorescence quantitative results of miRNA-126. **(B)** Real-time fluorescence quantitative results of miRNA-28-3p. **(C)** miRNA-126 and miRNA-28-3p correlation analysis. ^∗^*p* < 0.05.

After real-time fluorescence quantitative analysis of plasma miRNAs in two groups of patients, the expression level of miRNA-126 in plasma of DM patients (0.1162 ± 0.0236) was found to be higher than that of control patients (0.0018 ± 0.0862) and the difference was statistically significant (*P* < 0.01); the expression of miRNA-126 in the plasma of the DM group was ~60 times than that of the control group (Figure 1A). The expression level of miRNA-28-3p in the plasma of DM patients (0.1378 ± 0.0268) was higher than that in the control group (0.0006 ± 0.0167) and the difference was statistically significant (*P* < 0.01).

The expression of miRNA-28-3 in plasma of DM group was ~230 times in the control group (Figure 1B). Correlation analysis revealed a positive correlation between miRNA-126 and miRNA-28-3p (Figure 1C), with a Pearson correlation coefficient of 0.4337 and a 95% confidence interval (CI) of [0.2984, 0.5516] (*P* < 0.01).

### ROC Curve Analysis of miRNA-126 and miRNA-28-3p

**FIGURE 2:**
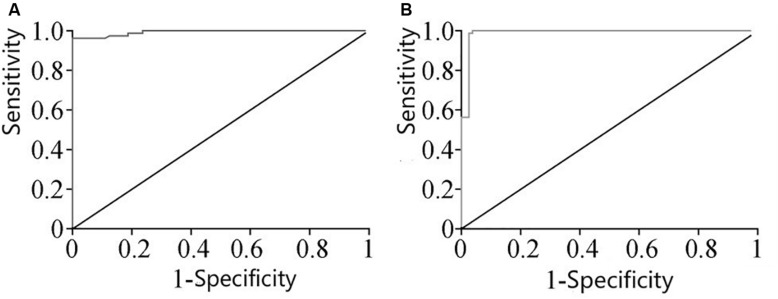
**(A)** ROC curve of miRNA-126 expression in plasma. **(B)** ROC curve of miRNA-28-3p expression in plasma.

The ROC curve analysis of miRNA-126 and miRNA-28-3p in plasma of the two groups of patients revealed that the area under the curve (AUC) of miRNA-126 in DM patients was 0.9989. The 95% CI was [0.984, 1.000], and there was a significant difference between the control group and the control group (*P* < 0.01). The miRNA-126 ROC curve is shown in Figure 2A, with a cut-off of 0.01124, a sensitivity of 96.35% (89.43–99.22%), and a specificity of 98.95% (93.23–99.97%); AUC of miRNA-28-3p ROC in DM patients was 0.9979, 95% CI was [0.9736, 1.000], which was significantly different from the control group (*P* < 0.01). Its ROC curve is shown in Figure 2B, with a cut-off value of 0.0020, a sensitivity of 99.35% (93.23–99.97%), and a specificity of 98.75% (91.26–99.70%).

### miRNA-126 and miRNA-28-3p Bioinformatics Analysis Results

**FIGURE 3:**
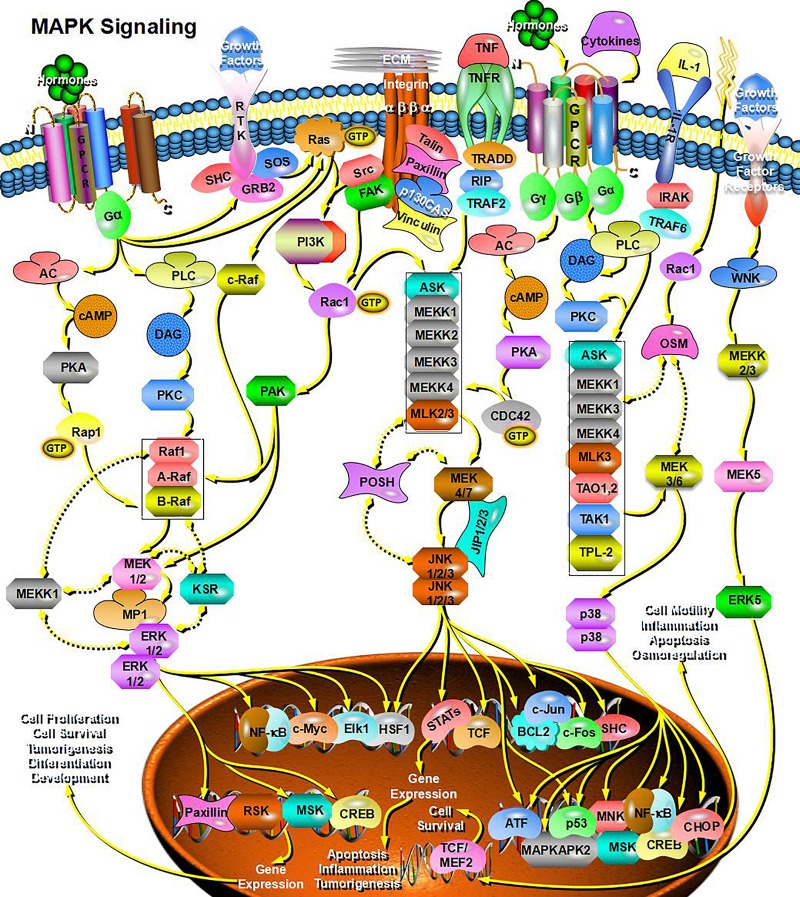
miRNA-28-3p promotes cell proliferation through the MAPK signaling pathway and participates in the regulation of diabetes.

**FIGURE 4:**
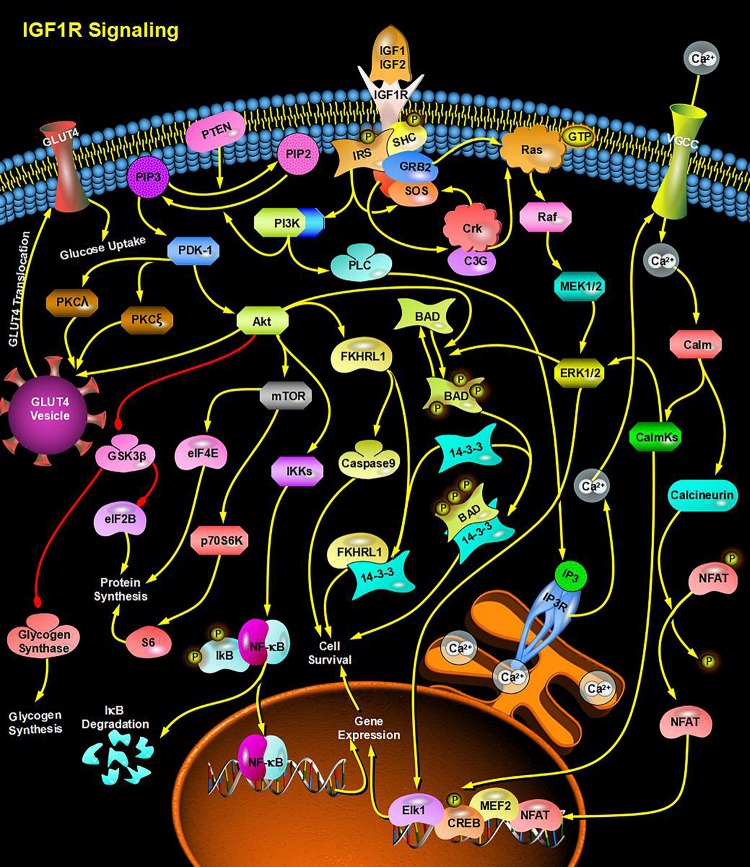
miRNA-28-3p promotes cell proliferation and migration through the IGF signaling pathway and participates in the regulation of diabetes.

**FIGURE 5:**
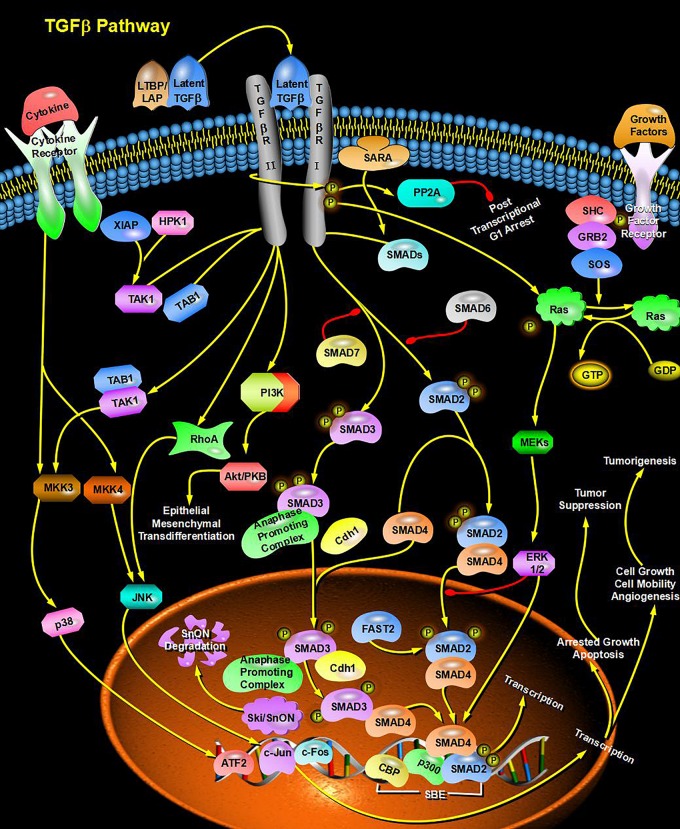
miRNA promotes signal transduction and glucose protein binding through the TGF-beta signaling pathway and participates in the regulation of diabetes.

**FIGURE 6:**
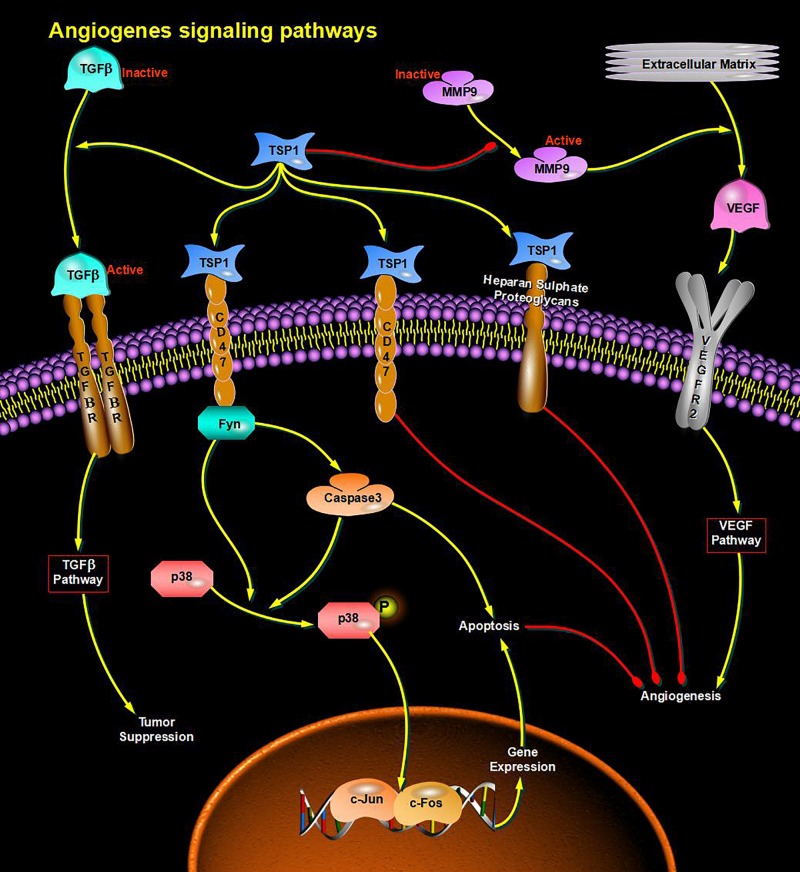
miRNA regulates cell proliferation and apoptosis via the Angiogenesis signaling pathway and participates in angiogenesis in diabetes.

Through bioinformatics analysis of multiple softwares, the target genes of miRNA-126 were found to be: VEGFA, SPRED1, PIK3R2, PARP16, TNFRSF11B, ADAM9, SLC7A5, and AKAP13; its biological functions are mainly associated with binding proteins, signal transduction, cell differentiation and regulation of cell morphology, etc.; its main role in regulating signaling pathways are: MAPK activation pathway, insulin receptor signaling pathway, insulin signaling pathway, chemokine signaling pathway, connexin signaling (Jak-STAT) pathways, and angiogenesis signal pathways. The target genes of miRNA-28-3p mainly include IGF1, IGF2R, MAP2K3, MAPK1, RAF1, RASA1, COIL, PSAP, CSNK1G1, MTHFD2L, C4orf52, BIRC5, SGK269, AP3M1, IKBKB, SLC9A8, MAPK1, DRAM1, CDKN1A, SRPK1, and BCL2L11, etc.; its biological functions are mainly related to cell proliferation, apoptosis, cell migration, signal transduction, glucose protein binding and fatty acid oxidative metabolism. Biological functions involved in the regulation of signaling pathways include: MAPK signaling pathway (Figure 3), insulin/IGF signaling pathway (Figure 4), endothelin signaling pathway, TGF-beta signaling pathway (Figure 5) and ngiogenesis signaling pathways (Figure 6).

**FIGURE 7:**
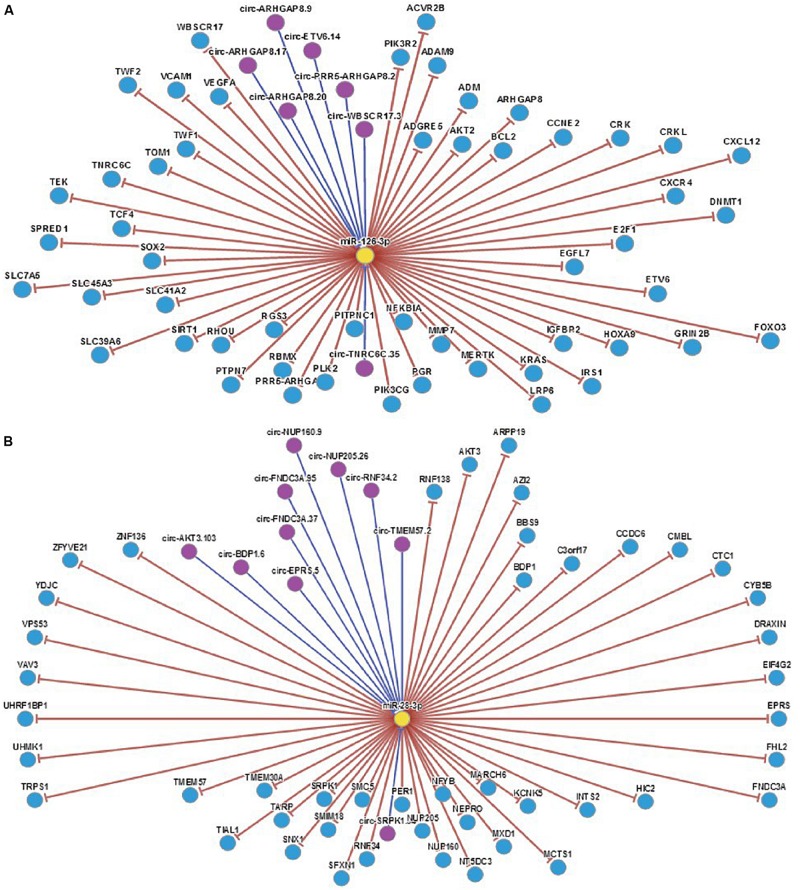
**(A)** Regulatory map of miRNA-126 related genes. **(B)** Regulatory map of miRNA-28-3p and related genes.

By analyzing the sequence structure of miRNA-126 and miRNA-28-3p, and predicting possible lncRNA and circRNA, it was found that the lncRNA related to miR-126 was XIST, and the circRNA was PLXNB2_hsa_circ_000681; the lncRNAs associated with miRNA-28-3p were RP11-197N18. 2, CTC-338M12. 3, OIP5-AS1, MMP24-AS1, RP11169K16. 9, CTD-2116N17. 1, RP11-815I9. RP11-384K6. 6, PLK1S1, AJ271736. 10, LOXL1-AS1, CTD-3014M21. 4, AC092535. 3, RP11-615I2. 7, RP4-647J21. 1, and XIST, and their related circRNA were ARL6IP4 _hsa_circ_000812, ITPR1 _ hsa _ circ _000705, C5orf25 _hsa_circ_001525, KIF2A_hsa_circ_001171, FKBP8_hsa_circ_ 000270, KDM3B_hsa_circ_001423, KIAA1586_hsa_ circ_0014 39, GBAS_hsa_circ_002070, STIM2_hsa_circ_001520, KPNB1_ hsa_circ_002147, HIST1H3D_hsa_circ_000744, CPNE1_hsa_ circ_000657, TIMELESS_hsa_circ_002140, FAM188A_hsa_circ_ 001836, FAM188A_hsa_circ_001349, FARSA_hsa_circ_000263, YLPM1_hsa_circ_001060 and CHEK2 _hsa_circ_001796, etc. Analysis of the relevant target genes and circRNAs revealed that complex genes and circRNAs are involved in the regulation (Figure 7). The interaction mechanism between miRNAs and circRNAs has been studied.

**FIGURE 8:**
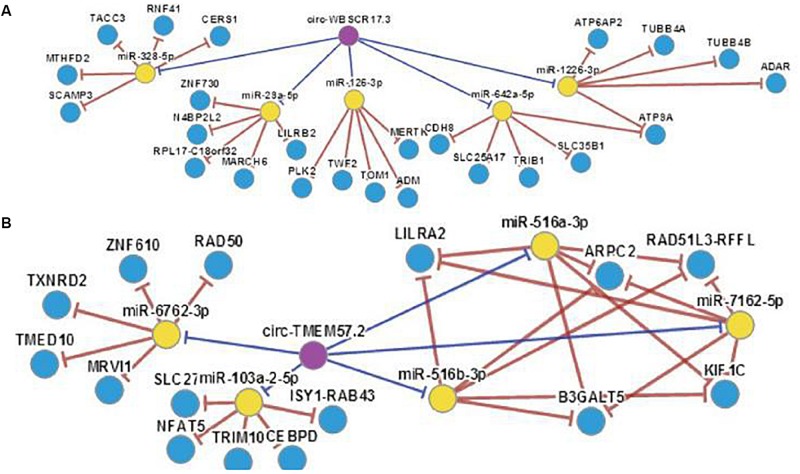
**(A)** miRNA-126 with associated “sponge” genes and circRNA. **(B)** miRNA-28-3p with associated “sponge” genes and circRNA.

**FIGURE 9:**
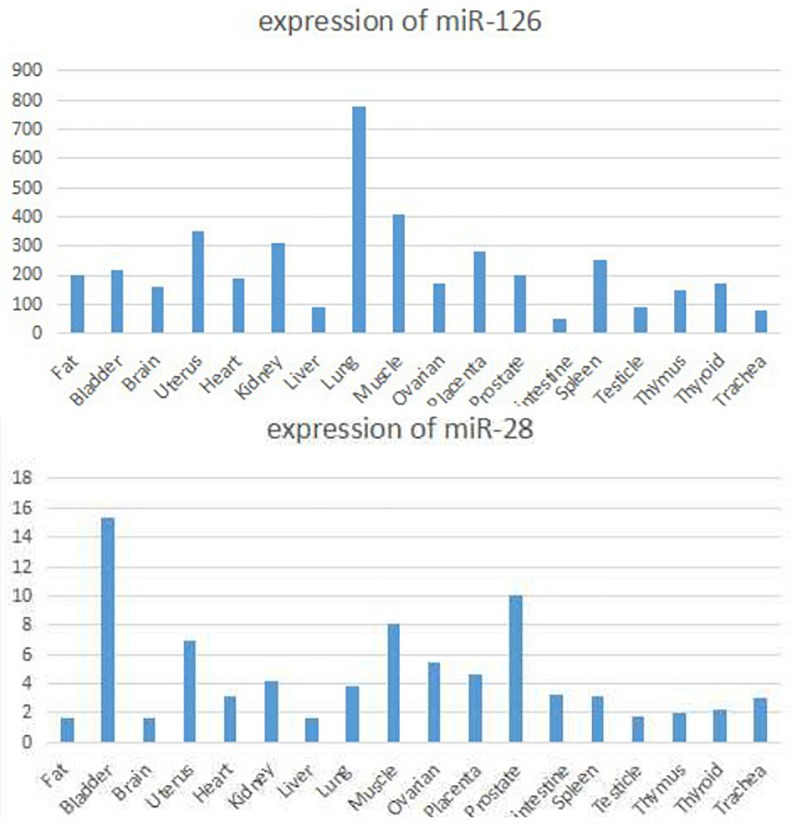
Organ specific expression of miRNA-126 and miRNA-28-3p.

Current studies suggest that circRNAs may be mainly used as “sponge” adsorption. They play a role in competitively inhibiting miRNA target genes, and we have discovered through software analysis a variety of miRNA126 and miRNA-28-3p sponge circRNAs and genes (Figure 8). At the same time, miRNA expression may have cell, tissue and organ specificity. We have found that miRNA-126 and miRNA-28-3p have relative expression in various organs through software enrichment analysis: miRNA-126 was found to be highly expressed in lung tissue, muscle tissue, and uterus, but was lower in liver and trachea tissues; miRNA-28-3p was expressed higher in the bladder, prostate, muscle, and uterus, but lower in liver, fat, and brain tissue (Figure 9). Researches on miRNAs did not simply remain at the level of miRNA expression. Most studies have performed in-depth functional studies, such as miRNA introns, exons, 3′-UTR, and related region polymorphism sites (SNPs). In other areas of research, we have found bioinformatics functional site distributions of miRNA-126 and miRNA-28-3p through bioinformatics analysis: the miRNA-126 functional region is mainly located on the EGFL7 and ve-statin genes on chromosome 9q12. Nearby, there are multiple SNP sites; miRNA-28-3p is mainly located near the LPP gene and has multiple SNP sites.

### Real-Time qPCR Verification of Target Genes

**FIGURE 10:**
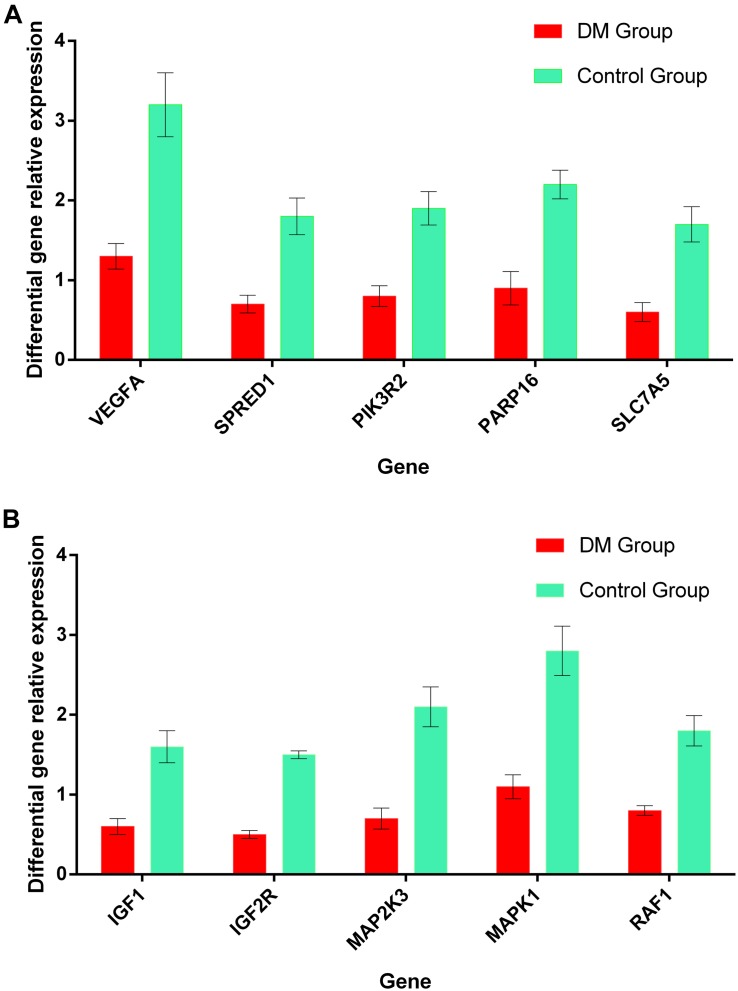
**(A)** The relative expression levels of the most relevant 5 target genes regulated by miRNA-126 in the plasma of DM and control group. **(B)** The relative expression levels of the most relevant 5 target genes regulated by miRNA-28-3p in the plasma of DM and control group.

Results of real-time quantitative PCR detection of differentially expressed genes were listed in Figure 10, the relative expression of each target gene in the figure was calculated according to the relative expression quantity = 2 – ΔCT formula, where ΔCT = CT value of target gene – CT value of internal reference gene (GAPDH). As can be seen from the figure, the expression level of all target genes in DM group was significantly lower than that in control group (Figure 10).

## Discussion

The number of people with DM in the world is large, and the population with high risk factors for DM has a large base. The prevention and treatment of DM are facing major challenges. In recent years, research on the pathogenesis of DM and miRNA has become a hot topic. Some miRNAs have also been found to be closely related to the pathogenesis of DM, especially in recent years, the discovery of cyclic miRNA and the maturity of its detection technology have opened up new avenues for DM research (Villard et al., 2015; Sebastiani et al., 2017). Extensive exonuclease is present in serum or plasma. Unmodified miRNAs are rapidly degraded in serum of plasma patients, and the stability of circulating miRNAs in serum or plasma is high, and they have strong ability against ribonuclease, so its detection has stronger reliability (Chen et al., 2014; Navickas et al., 2016). Wang et al. (2016b) found that the expression of miRNA-296 in plasma of DM patients was correlated with the plasma leptin level, while the expression of miRNA-9 was correlated with insulin levels; Yan et al. (2015) found that differentially expressed miRNAs in the plasma of DM and DM pre-patients, such as miRNA-1249, miRNA-320b, miRNA-572, and miRNA-6069, may be new biomarkers for early diagnosis of DM and potential therapeutic targets; Wang et al. (2014) conducted a study of Iranian and Swedish populations which found that plasma miRNA-93, miRNA-103, miRNA-191, miRNA-423-3p, miR-NA-425, and miRNA451 were all differentially expressed in DM patients and expressed in different populations. The situation is inconsistent; Pescador et al. (2013) found that both miRNA-15b and miRNA-376a may be involved in the pathogenesis of DM, and serum miRNA-503 and miRNA-138 may be used to identify obesity-induced and non-obesity-induced DM; another study showed that miR-143, miR-802, and miR-200a/b/c may cause DM through acting on mTORC2, GSK, and miR-103, miR-107, miR-221 and miR-223 may regulate PI3K-mTOR signaling pathway and cause DM to occur (Cheng et al., 2015; Chien et al., 2015; Mirra et al., 2015; Beermann et al., 2016). A preliminary study of our clinical sample found that the plasma miRNA-126 expression level in DM patients was significantly higher than that in the control group. The miRNA-126 expression in the DM group plasma was ~60 times higher than that in the control group. The 28-3p expression level in plasma of DM patients was about 230 times higher than that of the control group. This result suggests that miRNA-126 and miRNA-28-3p are significantly differently expressed in plasma of DM patients, and the expression of circulating miRNA-126 and miRNA-28-3p is positively correlated in the plasma of DM patients (Pearson correlation coefficient is 0.443), suggesting that miRNA-126 and miRNA-28-3p may be involved in the pathogenesis of DM.

With regard to the diagnosis of DM, the diagnosis is mainly based on the measurement of fasting blood glucose and postprandial blood glucose. However, there are many factors that interfere with blood glucose and are easily affected by the patient’s physical condition and diet. Therefore, it is necessary to actively explore other methods of diagnosis and treatment. We performed a ROC curve analysis of miRNA-126 and miRNA-28-3p in the plasma of two groups of patients and found that the AUC of miRNA-126 ROC was 0.993, and the cut-off value was 0.01124 with high sensitivity. 96.25%, specificity as high as 98.75%; AUC of miRNA-28-3p ROC was 0.998, when its cut-off value was 0.002038, its sensitivity was as high as 98.75%, and the specificity was as high as 97.50%, the results suggest that both miRNA-126 and miRNA-28-3p can be used as new biomarkers for potential clinical diagnosis of DM, and have higher sensitivity and specificity, providing a new approach for the diagnosis and treatment of DM.

In the final analysis, miRNA research still stays on the level of functional research. We first used bioinformatics analysis to find that both miRNA-126 and miRNA-28-3p may be involved in the insulin signaling pathway and the IGF signaling pathway (insulin/IGF pathway) and MAPK signaling pathways, and it can be speculated that miRNA-126 and miRNA-28-3p may cause DM through regulation of insulin signaling pathways and other related signaling pathways and target genes. Of course, this requires further functional testing and authenticating. Studies have shown that miRNAs interact with lncRNAs and circRNAs, etc. (Xing et al., 2014; Shi and David, 2015; Kong et al., 2016; Wang et al., 2016a), especially circRNAs, because of their high stability and specific expression ability, their research prospects are considerable, therefore, if combined miRNA, lncRNA, the research on the three circRNAs can provide a deeper understanding of the mechanism of disease and provide a better way for disease prevention and treatment. We found through bioinformatics analysis that both miRNA-126 and miRNA-28-3p regulate lncRNA-XIST and are associated with multiple circRNAs.

## Conclusion

In summary, both miRNA-126 and miRNA-28-3p may be involved in the pathogenesis of DM, and may serve as a novel biomarker for clinical diagnosis of DM. The main mechanism may be through regulation of insulin signaling pathway and insulin growth factor signaling pathway, and may be related to lncRNAs such as XIST and various circRNAs.

## Data Availability Statement

All datasets generated for this study are included in the article/supplementary material.

## Ethics Statement

This study was carried out in accordance with the recommendations of the Ethics Committee of the Second Affiliated Hospital of Nanchang University. The protocol was approved by the Ethics Committee of the Second Affiliated Hospital of Nanchang University. All subjects gave written informed consent in accordance with the Declaration of Helsinki.

## Author Contributions

HN: help to writing the manuscript and implementation of the experiment. KZ and KL: sample collection, blood testing, and analysis of data. JX: design research direction and writing manuscript. WZ: guidance article writing. ZF: review and revise the manuscript and guidance article writing.

## Conflict of Interest

The authors declare that the research was conducted in the absence of any commercial or financial relationships that could be construed as a potential conflict of interest.
